# Exclusive breastfeeding in rural Western China: does father’s co-residence matter?

**DOI:** 10.1186/s12889-021-12025-8

**Published:** 2021-11-02

**Authors:** Jingchun Nie, Lifang Zhang, Shuyi Song, Andrew John Hartnett, Zhuo Liu, Nan Wang, Weiqi Nie, Jie Yang, Ying Li, Yaojiang Shi

**Affiliations:** 1 Center for Experimental Economics in Education, Shaanxi Normal University, No. 620 West Chang’an Street, Chang’an District, Xian, Shaanxi Province China; 2 Business School, Beijing Normal University, Beijing, China; 3 Elliott School of International Affairs, George Washington University, Washington, D.C., USA

**Keywords:** Exclusive breastfeeding, Breastfeeding family support, father’s co-residence, Rural western China, Maternal decision-making power

## Abstract

**Background:**

China suffers from a low exclusive breastfeeding rate. Though it has been proofed that paternal support benefits breastfeeding a lot, the correlation between father’s co-residence and exclusive breastfeeding in China remain undiscovered. This study is to provide population-based evidence for the association of paternal co-residence on exclusive breastfeeding in rural western China. We also attempt to detect how the process works by examining the correlation between the father’s co-residence and breastfeeding family support as well as maternal decision-making power.

**Methods:**

A cross-sectional study was conducted in 13 nationally-designated poverty-stricken counties in the Qinba Mountains area in 2019. Data on breastfeeding practices, the status of fathers co-residence, breastfeeding family support, and maternal decision-making power were collected via structured questionnaires from 452 caregivers-infant pairs. Multivariate regressions were conducted to explore the correlation between paternal co-residence and exclusive breastfeeding.

**Results:**

The exclusive breastfeeding (0–6 months) rate was 16% in rural western China. Fathers’ co-residence was associated with a lower exclusive breastfeeding rate (*OR* = 0.413, 95% *CI* = 0.227–0.750, *P* = 0.004) and the rate did not improve when the father was the secondary caregiver. Even ruling out support from grandmothers, the association was still negative. Paternal co-residence did not improve maternal perceived breastfeeding family support, neither practically nor emotionally (*β* =0.109, *P* = 0.105; *β* =0.011,*P* = 0.791, respectively) and it reduced maternal decision-making power (*β* = − 0.196, *P* = 0.007).

**Conclusions:**

Fathers’ co-residence is negatively associated with the exclusive breastfeeding rates in rural western China. More skill-based practical and emotional strategies should be considered on father’s education to help them better involvement and show more respect to mothers’ decisions.

## Background

Compelling evidence shows that breastfeeding provides numerous benefits for both infants and mothers, including fewer infections for infants and less chance of breast cancer, ovarian cancer and type 2 diabetes for mothers [1]. Many countries including China cite the improvement of breastfeeding as a major public health issue [2]. However, China still suffers from a low exclusive breastfeeding rate for the first 6 month ranging from 20 to 30% in both urban and rural areas [3, 4]. With a growing number of women being employed, inadequate paid maternity leave and unsupportive work environments are major contributors to the low exclusive breastfeeding rate worldwide [5, 6]. However, mothers in rural areas either cease to migrate to cities for works or being unemployed during the first few months of newborns,^1^ which enables mothers to stay with infants all the time and feed them on demand. So the low exclusive breastfeeding rate must be caused by some other factors. Further, 138 million children live in rural China and nearly half of them are in poor areas [7]. Understanding the exclusive breastfeeding practice in rural China are essential for those children to get adequate nutrition, and for Chinese government to achieve the exclusive breastfeeding rate target of 50% by 2020 [8].

Determinants of breastfeeding have been studied widely, such as caesarean section rate, maternal education, family income and paternal involvement [4, 9–11]. Of these influential factors, paternal support is less discussed in the context of China rural areas, where a majority of fathers are rarely co-resident in the household but migrating to cities for work. Dozens of previous researches have documented the importance of paternal support on successful breastfeeding practice [12–15]. Paternal practical support includes taking care of infants and assisting with household chores, such as helping to burp the baby, changing diapers, washing clothes, and is quite supportive for earlier breastfeeding initiation and longer breastfeeding duration [12, 16]. Paternal emotional support, including encouragement, compliments, affection and reassurance at difficult moments, is also an essential ingredient to successful breastfeeding, which increases mother’s confidence and enables her to maintain an adequate milk supply [10, 17, 18].

Barrier of providing paternal support may exist in rural areas of China where fathers are not co-resident with their spouses. Statistics showed that male labors fluxed to urban areas have reached 79.18 million in 2018 in this area, accounting for 28% of the total national migrate labor [19]. More than half of the children (56%) under 3 years old live in western rural areas with their fathers being absent [20]. Therefore, we select rural western China to explore the association of father’s co-residency and exclusive breastfeeding rate.

The assumption that father’s co-residence is positively associated with exclusive breastfeeding may oversimplify the situation. Though fathers will have more chance to take care of mothers and infants when they are living at home, they may rarely do so. On one hand, since fathers are shouldering the main financial responsibility of the whole family, they need to do farm work as well as some part-time job into cover the daily expense which may make them too exhausted to provide their support on breastfeeding [21]. On the other hand, old generation, particularly the infant’s grandmothers, are regarded as a main source of breastfeeding family support, especially when lacking of learning materials and community consultants [22–24]. However, father’s co-residence may crowd out the support from grandmothers who are more experienced, thus impair the exclusive breastfeeding practices. Moreover, research suggests that fathers in rural areas tend to be low-educated and less knowledgeable about exclusive breastfeeding [25–27] and less adept at comforting mothers in feeding problems or expressing encouragement and praise for mother’s greatness due to gender norms [21]. To clarify these guesses, it is important to examine the association of father’s co-residence on exclusive breastfeeding. Limitations also exist in methodology of previous studies about exclusive breastfeeding in rural China, such as small and convenience sample instead of representative sample sizes [17, 28, 29]. Therefore, population-based evidence from rural China is crucial to bridge this gap.

Apart from providing breastfeeding family support, father’s co-residence may have an additional impact on reducing maternal decision-making power, which is examined as a determinant of infants health and nutrition [30, 31]. In rural areas of China, women are still in a relatively weak position in terms of family decision-making [32, 33]. It is even more so when fathers were involved in baby care. However, evidence showed that women with more decision-making power were associated with longer breastfeeding duration, more dietary diversity and less child stunting [31, 34, 35], which can help improve infants’ health and nutrition status.

Above all, the objective of this study is to provide population-based evidence of the influence of father’s co-residence on exclusive breastfeeding in western rural China. Furthermore, we try to understand how the process works by examining the correlation of the father’s co-residence on breastfeeding family support and maternal decision-making power. This study will enrich literature on the relationship between father’s co-residence and exclusive breastfeeding, thereby raising important implications for public policy to encourage fathers’ involvement in parenting and to promote exclusive breastfeeding in rural China.

## Methods

### Setting

This study was conducted in two prefecture-level cities from the Qinba Mountains area. The Qinba Mountains area spans six provinces of western China located in Qinling and Bashan Mountains, which is one of the eight multidimensional Poverty in Regions of Contiguous Distress [36]. At the end of 2018, there was 1.97 million poverty-stricken population in this entire region which accounted for 11.9% of the national population of poor people, and 67 nationally-designated poverty-stricken counties which comprised 11.4% of the nation total poverty-stricken counties in China [36, 37]. Within the Qinba Mountains area, nearly half of them live in the countryside [37]. Our data collection was conducted in 13 nationally-designated poverty-stricken counties from two prefecture-level cities in this area.

### Sample

The inclusion criteria of the study were: (a) the infant had been a resident of the sample village after delivery; (b) the infant was aged between 0 and 6 months (0 to 180 days) old. The exclusion criteria were: (a) the primary caregiver was not the mother; (b) the primary caregiver had cognitive impairment; (c) parents of the infant divorced or at least one parent died. We recognized those as cognitive disorders who cannot understand our structural questionnaire after repeating for three times and checked the condition with their family. Respondents included the primary caregiver (mother) and secondary caregiver (the person who spends the most time and efforts in taking care of the infants except for the primary caregiver, usually the father or grandparent) of the infant.

The sample size was estimated to achieve, for a binomial variable of 0.15 in consideration of our pilot finding, a standard error of 0.025 with a 95% confidence interval of 0.10–0.20. The final desired sample size was 430.

Cluster random sampling was used to select participants following three steps. First, we got the village list from local health commissions and randomly selected half of the rural villages from each of the 13 counties, which constitute our initial sample frame of 876 villages. Then we acquired the newborn list from local health commissions and checked the list with the primary healthcare providers in each village, who are responsible for managing the newborns in the village. We excluded villages with fewer than 3 or more than 15 eligible participants with a comprehensive consideration of research costs and power requirements, after which 202 villages were eligible to be included in final sample frame. We used Stata 15.0 (Stata Corp, College Station, TX) to generate random numbers and obtained 131 sample villages from the eligible villages based on the sample size calculation. Finally, in each of the 131 eligible villages, all caregivers-infant pairs meeting our inclusion criteria above were included, thus constituted a total number of 467 eligible caregivers-infant pairs representing Qinba Mountains area. Of these, 10 caregivers-infant pairs were excluded because mothers were not the first caregivers of infants and 5 were excluded because of missing data. Finally, 452 caregivers-infant pairs were included in the analysis.

### Data collection

Data was collected in March 2019. With the assistance of the local healthcare provider in each village, a consent form with information about program objectives, procedure, potential risks and benefits, as well as privacy protection was distributed to each eligible participant before the face-to-face interview by one enumerator. To ensure accuracy and consistency during the procedure of data collection, the enumerators were trained intensively, followed by a simulation exercise with twenty participants prior to data collection. The questions were shown in tablet and asked one by one by the enumerator while answers recording timely. After the interview with the mother, a short questionnaire was given to the secondary caregiver as well. Each interview was conducted alone to avoid interruptions from others.

### Measurements

#### Demographics

Demographic information was collected from the primary caregiver (the mother) about the infant’s gender, age (based on the number of days), method of delivery, small for gestation age (SGA), preterm delivery (gestational age < 37 weeks) or not, as well as other personal information about the caregiver including maternal age and education level, number of siblings and the ownership of nine selected family asset items. Caregivers’ nutrition and health knowledge was also assessed according to their responses on eight questions about infant’s health and the benefits of exclusive breastfeeding. We calculated the total score as a measurement of their nutrition and health knowledge. We also asked who the secondary caregiver of the infant was.

*Father’s co-residence* is the independent variable of interest. It was coded as “1” if the father living at home when the survey was conducted, and “0” if the father is absent.

*Exclusive breastfeeding* is the main outcome of interest. The feeding practices of infants within the last 24 h before the survey were recorded, including whether the infant was fed breastmilk, formulas, fresh animal milk, water, juice, etc. Exclusive breastfeeding was defined as that the infant receives only breastmilk, and no other liquids or solids are given (not even water) [1].

*Breastfeeding family support* is one of the intermediate outcomes. Breastfeeding family support was measured using a scale designed by Zhu Xiu (2013) which contained 9 items. The Cronbach’ alpha value of the scale was 0.886, suggesting adequate internal consistent reliability [38]. For each item, participants were asked to rate their level of agreement on a 5-point Likert-type scale ranging from strongly disagree to strongly agree. This scale can be divided into two dimensions: practical support which include 2 items (such as “my family often prepares food that is good for lactation”) and emotional support which include 7 items (such as “I think my family wants me to exclusively breastfeed my child”). We calculated the mean score of each dimension and the higher score indicates stronger perceived family support.

*Maternal decision-making power* is another intermediate outcome*.* Maternal decision-making power was measured by the scale adapted from Peterman et al.(2015) and Shroff et al.(2011) [35, 39, 40]. The Cronbach’ alpha value of this scale was 0.604, suggesting an acceptable internal consistent reliability in evaluating decision-making power according to Nunnally and Bernstein (1994) [39]. We asked who had the final say on eight household decisions about childcare and household consumption in the family. Options included “respondent”, “jointly with others in the household” and “others in the household”. Instead of using the raw score total, we coded the answer of “respondent” as “1″ and others as “0″. Then we referred to Shroff et al. (2011) and conducted a factor analysis to generate an index indicating the level of maternal decision-making power [35]. A higher score indicated that mother was more powerful in making decision by herself.

### Data analysis

All analyses were performed using Stata 15.0. Descriptive analysis was presented as number (%) for categorical variables and mean (*SD*) for continuous variables. We performed student’s *t-tests* and reported *p-values* to assess the unadjusted association between father’s co-residence and exclusive breastfeeding, breastfeeding family support and maternal decision-making power. Multivariable logistic regressions were used to explore the correlation of the father’s co-residence and exclusive breastfeeding, while odds ratios (*ORs*) and corresponding 95% confidence intervals (*CIs*) were reported as a measurement of the risk. We also performed multivariable linear regressions to estimate the correlation of father’s co-residence and breastfeeding family support (including practical and emotional support respectively) and maternal decision-making power. Coefficient and corresponding 95% confidence intervals were also presented. All multivariable regressions controlled the infants’ characteristics (age, sex, method of delivery, SGA, whether preterm), maternal characteristics (age, education level and nutrition and health knowledge), family characteristics (number of siblings and family asset index) and county fixed effects. The 0.05 nominal level of significance was used for all statistical tests.

## Results

In total, 452 family-infant pairs were included in our final analysis after excluding 15 (3.2%) pairs with missing values. Basic characteristics of the participants and their correlations with father’s co-residence were reported in Table 1. Specifically, there were more than half of the fathers (53.5%) co-residing with their families. The mean age of the 452 infants was 94.0 days (range: 6 to 180 days), among which 52.9% (239) were male. Besides, more than 60% of the infants had one or more siblings. Participated mothers were 28.3 years old on average (range: 18 to 45 years). In terms of educational level, 50.2% (228) of the mothers stopped at junior high, and 27.1% (122) of them had completed senior high school or above. The mean score of maternal nutrition and health knowledge was 4.5 (*SD* = 1.5) out of 8, meaning that caregivers in rural areas were lack of knowledge about breastfeeding. The exclusive breastfeeding rate was significantly lower in the subgroup of father’s co-residency than that of father’s non-co-residence (11% compared to 22%, *P* < 0.01). Besides, 40.2% (97) of co-resident fathers were secondary caregivers, and less grandmothers were secondary caregivers because of father’s co-residence (45% compared to 64% of father’s non-co-residence, *P* < 0.01). There was no significant difference in terms of mother’s perceived breastfeeding family support, neither practical nor emotional, between the two subgroups, while a significant difference did exist in maternal decision-making power (*t* = 2.86, *P* = 0.004).

**Table 1:** Characteristics of infants living with or without fathers

Characteristics	(1)	(2)	(3)	(4)
	Full sample n1 = 452 No.(%) or Mean(SD)	Living with father n2 = 242 No.(%) or Mean(SD)	Living without father n3 = 210 No.(%) or Mean(SD)	*P-*value
**Infants’ characteristics**				
Infants’ age (day) ^a^	94.0 (±51.2)	86.5 (±55.7)	102.4 (±44.1)	**0.001**
Male	239 (52.9%)	124 (51.2%)	115 (54.8%)	0.456
Caesarean section	200 (44.4%)	106 (44.0%)	94 (45.0%)	0.838
Small for gestational age	25 (5.6%)	17 (7.1%)	8 (3.8%)	0.136
Preterm	24 (5.3%)	15 (6.2%)	9 (4.3%)	0.367
**Maternal and family characteristics**				
Maternal age ^a^	28.3 (±4.63)	28.5 (±5.00)	28.0(±4.23)	0.278
Maternal education				**0.008**
Primary school and below	102 (22.7%)	66 (27.4%)	36 (17.2%)	
Junior high school	228 (50.2%)	108 (44.6)	120 (57.1%)	
Senior high school and above	122 (27.1%)	68 (28.2%)	54 (25.8%)	
Maternal nutrition and health knowledge ^a^	4.48 (±1.45)	4.33 (±1.41)	4.66 (±1.49)	**0.020**
Number of siblings				**0.031**
0	165 (36.5%)	74 (30.6%)	91 (43.3%)	
1	255 (56.4%)	151 (62.4%)	104 (49.5%)	
2 or more	32 (7.1%)	17 (7.02%)	15 (7.15%)	
Family asset index ^a^	−0.005 (±1.06)	0.049 (±1.09)	− 0.068 (±1.02)	0.242
**Dependent and independent variables**				
Exclusive breastfeeding	72 (15.9%)	26 (10.7%)	46 (21.9%)	0.001
Father functions as secondary caregiver	97 (21.5%)	97 (40.2%)	0	**< 0.001**
Grandmother functions as secondary caregiver	242 (53.5%)	108 (44.6%)	134 (63.8%)	**<** 0.001
Breastfeeding family support				
Practical support ^a^	3.89 (±0.70)	3.94 (±0.66)	3.83 (±0.74)	0.106
Emotional support ^a^	3.57 (±0.40)	3.57 (±0.40)	3.57 (±0.41)	0.992
Index of maternal decision-making power ^a^	0.004 (±0.75)	−0.09 (±0.75)	0.11 (±0.73)	**0.004**

^a^Data are reported as “mean (SD)”

According to the multivariable regression results presented in Table 2, father’s co-residence was significantly and negatively associated with exclusive breastfeeding (*OR* = 0.413, 95% *CI* = 0.227–0.750, *P* = 0.004; column 1), which was consistent with the descriptive analysis. Mothers with fathers’ co-residence were further split into two subgroups based on whether fathers function as secondary caregivers or not. Compared with the mothers without fathers’ co-residence, the two subgroups of mothers with fathers’ co-residence both had significantly lower rate of exclusive breastfeeding (OR = 0.397, 95% CI = 0.173–0.912, *P* = 0.029; OR = 0.423, 95% CI = 0.211–0.848, *P* = 0.015, respectively; column 2), while the odd ratios of the two subgroups were very close (*P* = 0.830). Even grandmother functions as secondary caregiver was taken into consideration, the correlation of father’s co-residence and exclusive breastfeeding was still significantly negative (*OR* = 0.395, *95% CI* = 0.216–0.724, *P* = 0.003; column 3).

**Table 2:** Multiple Logistic Regression of Father’s Co-residence on Exclusive Breastfeeding *(N = 452)*

Dependent variable: exclusive breastfeeding	(1) Fathers’ co-residence		(2) Fathers’ involvement when co-reside		(3) “Crowd out” effect	
	*OR* (*95%CI*)	*P Value*	*OR* (*95%CI*)	*P Value*	*OR* (*95%CI*)	*P Value*
Father’s co-residence (1 = yes)	0.413 (0.227–0.750)	0.004			0.395 (0.216–0.724)	0.003
Father functions as secondary caregiver			0.397 (0.173–0.912)	0.029		
Father not functions as secondary caregiver			0.423 (0.211–0.848)	0.015		
Grandmother functions as secondary caregiver					0.752 (0.413–1.370)	0.351
**Infants’ characteristics**						
Infants’ gender (1 = male)	1.140 (0.643–2.023)	0.654	1.139 (0.642–2.021)	0.657	1.153 (0.649–2.050)	0.628
Infants’ age (day)	0.994 (0.988–1.000)	0.046	0.994 (0.988–1.000)	0.045	0.994 (0.998–1.000)	0.048
Caesarean section (1 = yes)	0.682 (0.375–1.242)	0.211	0.683 (0.375–1.244)	0.213	0.663 (0.363–1.212)	0.182
Small for gestational age (1 = yes)	2.244 (0.548–9.186)	0.261	2.240 (0.547–9.178)	0.262	2.390 (0.581–9.843)	0.227
Preterm (1 = yes)	0.295 (0.051–1.720)	0.175	0.298 (0.051–1.751)	0.180	0.271 (0.046–1.595)	0.149
**Maternal and family characteristics**						
Maternal age	0.960 (0.887–1.040)	0.317	0.961 (0.887–1.042)	0.335	0.952 (0.877–1.033)	0.235
Maternal education (Primary school and below as reference)						
Junior high school	0.795 (0.361–1.752)	0.570	0.798 (0.361–1.761)	0.576	0.799 (0.363–1.757)	0.576
Senior high school and above	0.640 (0.253–1.619)	0.346	0.641 (0.253–1.624)	0.348	0.644 (0.254–1.633)	0.354
Maternal nutrition and health knowledge	1.447 (1.173–1.785)	0.001	1.448 (1.173–1.788)	0.001	1.431 (1.159–1.768)	0.001
Number of siblings	1.363 (0.792–2.345)	0.264	1.362 (0.791–2.344)	0.265	1.347 (0.783–2.319)	0.282
Family asset index	1.149 (0.839–1.573)	0.386	1.147 (0.836–1.573)	0.396	1.152 (0.842–1.575)	0.377

All regressions include county fixed effect

We used some intermediate variables to further analyze why there was a negative impact of father’s co-residence on exclusive breastfeeding, and results were shown in Table 3. Our data indicated that the father’s co-residence was not associated with either maternal perceived practical support (*β* =0.109, *95% CI* = -0.023–0.241, *P* = 0.105) or emotional support (*β* =0.011, *95% CI* = -0.068–0.090, *P* = 0.791). Nevertheless, mothers would have less decision-making power in family decisions when they co-reside with a father (*β* = − 0.196, *95% CI* = -0.339- -0.053, *P* = 0.007), which was also consistent with the descriptive analysis.

**Table 3:** Multiple Linear Regression of Father’s Co-residence on Intermediate Variables: Breastfeeding Family Support and Decision-Making Power *(N = 452)*

Dependent variables	(1) Practical support		(2) Emotional support		(3) Index of maternal decision making power	
	Coefficient (*95%CI*)	*P Value*	Coefficient (*95%CI*)	*P Value*	Coefficient (*95%CI*)	*P Valve*
Father’s co-residence (1 = yes)	0.109 (−0.023–0.241)	0.105	0.011 (−0.068–0.090)	0.791	− 0.196(− 0.339- -0.053)	0.007
**Infants’ characteristics**						
Infants’ gender (1 = male)	−0.018 (− 0.145–0.110)	0.784	0.003 (− 0.073–0.080)	0.931	−0.012 (− 0.150–0.126)	0.861
Infants’ age (day)	−0.001 (− 0.003 --0.000)	0.026	−0.001 (− 0.001–0.000)	0.151	0.002 (0.000–0.003)	0.010
Caesarean section (1 = yes)	− 0.022 (− 0.151–0.107)	0.737	0.025 (− 0.052–0.103)	0.519	−0.160 (− 0.300- -0.020)	0.025
Small for gestational age (1 = yes)	−0.183 (− 0.494–0.128)	0.248	−0.063 (− 0.248–0.123)	0.508	−0.252 (− 0.588–0.084)	0.142
Preterm (1 = yes)	− 0.059 (− 0.262–0.380)	0.718	−0.102 (− 0.090–0.294)	0.296	0.023 (− 0.370–0.325)	0.898
**Maternal and family characteristics**						
Maternal age	0.000 (− 0.016–0.016)	0.962	−0.005 (− 0.014–0.005)	0.329	0.000 (− 0.017–0.018)	0.956
Maternal education (Primary school and below as reference)						
Junior high school	0.101 (− 0.078–0.281)	0.268	0.017 (− 0.091–0.124)	0.763	−0.049 (− 0.244–0.145)	0.618
Senior high school and above	0.204 (− 0.009–0.417)	0.061	0.021 (− 0.107–0.148)	0.751	−0.020 (− 0.250–0.211)	0.865
Maternal nutrition and health knowledge	0.057 (0.010–0.103)	0.017	0.039 (0.012–0.067)	0.005	0.023 (−0.027–0.073)	0.362
Number of siblings	−0.071 (− 0.194–0.051)	0.254	−0.009 (− 0.083–0.064)	0.805	0.197 (0.064–0.330)	0.004
Family asset index	− 0.017 (− 0.084–0.051)	0.624	−0.057 (− 0.097 --0.017)	0.006	0.007 (− 0.066–0.080)	0.851

All regressions include county fixed effect

## Discussion

This study reports results from a population-based survey examining the influence of father’s co-residence on exclusive breastfeeding in western rural China. Our findings showed that the exclusive breastfeeding rate was as low as 16% in rural China, indicating a large gap from the target of 50% in 2020 set by Chinese government. When adopted the measurement of predominant breastfeeding, that is, infants who were fed by human milk and water [9], the rate was 42%. Infant’s age and maternal nutrition and health knowledge were significantly associated with exclusive breastfeeding, which was consistent with the existing research [9, 11, 41] while other factors such as caesarean section, maternal education and maternal age not. More attention and greater effort are still needed to promote exclusive breastfeeding in western rural China.

Our findings indicate that father’s co-residence was significantly negatively associated with exclusive breastfeeding, even when endogenous explanations, such as infants’ age, are taken into consideration. Though some literature from low- and middle-income countries, such as India, documented no correlation between fathers’ involvement and better breastfeeding [42], our finding of negative correlation is still interesting and contrary to our assumption. Under the encouragement of governments’ policies, more migrants are inspired to return home and start their own business, hence fathers are expected to have more chance to co-reside with mothers in the future. Therefore, there is a need to go beyond the aggregate results to examine possible explanations. However, to our knowledge, there are few literatures on the correlation of father’s co-residence and mothers’ breastfeeding behavior except for the study of Emmott & Mace (2015). Our findings are consistent with the study of Emmott & Mace (2015) which conducted in the UK. It documented that the involvement of household chores was related with higher risk of breastfeeding termination [43].

It may because fathers are rarely involved in taking care of infants that cause the negative impact on breastfeeding, since whether fathers migrate to work or not, they carry the financial burden of the entire family and have little time to take care of the mother and infant [44]. Obviously, fathers of co-residence who function as the secondary caregivers will have more opportunity to provide family support than those do not. However, both of the subgroups have a significantly lower and almost the same rate of exclusive breastfeeding than that of father’s non-co-residence, indicating a not reasonable explanatory for our findings.

Another explanation may be the “crowd out” effect, that is, father’s co-residence may crowd out or replace the support from other family members (such as the grandmothers). The finding that less grandmother functions as the secondary caregiver when father co-reside in the household (45% vs. 64%, *t* = 4.15, *P* < 0.01) indicated that the “crowd out” effect do exist, which helped us to understand why the exclusive breastfeeding practices did not change significantly when fathers didn’t co-reside. However, the magnitude of coefficient of father’s co-residence on exclusive breastfeeding was still negative and almost the same after controlling grandmothers as the secondary caregivers. Hence, the “crowd out” effect is also not the proper reason for the negative impact of father’s co-residence.

Our findings also indicate that father’s co-residence has no significant impacts on maternal perceived breastfeeding family support, neither practically nor emotionally, which may have two implications. On one hand, mothers don’t feel supportive from fathers. This may partly because fathers are less knowledgeable about exclusive breastfeeding and don’t know how to assist with breastfeeding challenges [14, 44, 45]. In our sample, the average nutrition and health knowledge score of fathers who function as secondary caregivers was 3.64 (*SD* = 1.26), which was significantly lower than that of the mothers’ (*M* = 4.48, *SD* = 1.45, *P* < 0.01). On the other hand, though few improvements are made, paternal support is not too bad or detrimental to decrease the rate of exclusive breastfeeding. Therefore, paternal support is somewhat explanatory but not sufficient for explaining our findings.

Could there exist an additional explanation besides paternal support? The support and resource for successful exclusive breastfeeding should be either from (1) mothers passively receive from the family and society, such as paternal support, or (2) mothers actively seek by themselves. The mother’s ability to actively seek support and resource, measured by decision-making power in our context, should be essential while the former one alone is not enough. Our data showed the exclusive breastfeeding rate was 19% when the index of maternal decision-making power was above the mean, and it significantly decreased to 11% when the mother was less powerful. Multiple logistic regressions also revealed significantly positive relation between maternal decision-making power and exclusive breastfeeding (Appendix Table 1). The possible and direct explanation is that mothers with lower decision-making power will be less capable to reject the incorrect feeding practices and opinions from fathers. This power for mother will be particularly important when father is not such supportive which we had shown above. Besides, there are also some possible indirect explanations for this finding. First, mothers with lower decision-making power are usually associated with less accessible to necessary resource for better breastfeeding, such as breastfeeding skill trainings or professional consulting. Moreover, less decision-making power is usually associated with less maternal motivation or even more mental health problem [35], which will hinder the mother to overcome the challenges associated with breastfeeding, such as continuing breastfeeding when encounter breast pains. Finally, unlike making decisions alone, more participants in a decision-making process may follow with more time to make decision, more disagreements and more inconsistent actions after decision, which may lead to worse consequence [6, 46]. Additional research is need to exam the above possible explanation.

### Limitations

There are several limitations in this study. First, only breastfeeding family support are assessed in this study and we did not interview fathers directly, which limit our understanding of how fathers involve in breastfeeding and interact with mothers privately. Besides, though we are trying to give some reasonable explanations to identify how maternal decision-making are associated with exclusive breastfeeding, more in-depth studies are required to better understand the interpersonal dynamics between couples. Second, all samples come from rural areas of western China, which limits external validity with reference to other segments of the population. Finally, we limit the definition of exclusive breastfeeding in our study. As this is a cross-sectional study, exclusive breastfeeding in our study is assessed based on the practices in the last 24 h prior to the survey date instead of within the first 6 month after delivery as WHO recommends. However, our measurement of exclusive breastfeeding is also recommend by WHO [1], so we would not expect this measurement to lead to bias in our conclusions.

## Conclusions

This study found that co-residence with fathers was associated with a significantly lower rate of exclusive breastfeeding in rural western China. Father’s co-residence did not improve maternal perceived family support, neither practically nor emotionally. Besides, mother’s decision making will become more difficult because of father’s involvement. It implies that the low rate of exclusive breastfeeding cannot be attributed to the father’s absence, although the father’s migration to work in urban areas is common in rural western China.

The findings from this study have important implications for public health policy and practice. Only father’s co-residence with the mother is not enough to improve exclusive breastfeeding in rural China. Education aimed at fathers in rural China should be considered and breastfeeding programs should consider the involvement of fathers in the future [47]. On one hand, fathers need more skill-based training on practical information regarding the benefits, process, and management of breastfeeding problems, as well as emotional-targeted strategies like how to express affection, encouragement and admiration to their partner, to provide high quality support for mothers [21, 48–50]. On the other hand, as it is the mother who provides breastfeeding for the infant, they should be fully respected and protected to make their own decisions during lactation.

## Data Availability

The datasets used and analyzed during the current study are available from the corresponding author on reasonable request.

## Appendix

**Table 4:** Multiple Logistic Regression of Maternal Decision-Making Power on Exclusive Breastfeeding *(N = 452)*

Dependent variable: exclusive breastfeeding	Coefficient (*95% CI)*	*P Value*
Index of maternal decision-making power	0.437 (0.041–0.834)	0.031
**Infants’ characteristics**		
Infants’ gender (1 = male)	0.113 (− 0.453–0.680)	0.695
Infants’ age (day)	−0.006 (− 0.011–0.000)	0.060
Caesarean section (1 = yes)	−0.270 (− 0.864–0.324)	0.373
Small for gestational age (1 = yes)	0.821 (− 0.575–2.217)	0.249
Preterm (1 = yes)	−1.416 (−3.209–0.378)	0.122
**Maternal and family characteristics**		
Maternal age	−0.051 (− 0.130–0.028)	0.209
Maternal education (Primary school and below as reference)		
Junior high school	− 0.078 (− 0.855–0.700)	0.845
Senior high school and above	−0.362 (− 1.283–0.560)	0.442
Maternal nutrition and health knowledge	0.387 (0.178–0.595)	0.000
Number of siblings	0.155 (− 0.386–0.695)	0.575
Family asset index	0.075 (− 0.231–0.382)	0.630

The regression includes county fixed effect

## Footnotes

1 In our ample, over 90% mothers’ main job is to look after their infants currently; others, need to do some housework, but no one is employed at that time.
